# Cutting piles in Crohn's disease

**DOI:** 10.1007/s10151-022-02571-7

**Published:** 2022-02-10

**Authors:** Andrew Zbar

**Affiliations:** 1 Department of Anatomy and Neuroscience, The University of Melbourne, Melbourne, Australia; 2 Department of Anatomy and Physiology, The University of Melbourne, Melbourne, Australia

**Keywords:** Haemorrhoidectomy, Crohn's disease, Proctectomy

Dear Sir,

I would broadly agree with the principles expressed by Alam et al. [1] concerning the decision to perform haemorrhoid surgery in patients with Crohn’s disease and the surgical technique used. Most experienced coloproctologists, however, will have their repository of Crohn’s disease patients where they have managed symptomatic haemorrhoids successfully with usually some modified version of a conventional ligation-excision procedure. When performed where indicated as a locally isolated operation for a single area of symptomatic prolapse often with submucosal excision (in patients without rectal or perianal disease or when their pre-existent rectal disease is completely inactive), the short- and long-term results are certainly acceptable. This personal clinical impression is backed up with extensive follow-up from Wolkomir and Luchtefeld showing the safety of such a selective approach [2].

One of the reasons why haemorrhoidectomy in Crohn’s disease has such a poor reputation is historical. When Jean Ritchie and colleagues reported the St. Mark’s results in 1977, they were disturbing [3]. Of 20 patients with Crohn’s disease in the 12 operated for haemorrhoids, half ended up with a proctectomy mostly for severe postoperative perirectal sepsis. The 1977 paper, however, is worth re-reading. The authors retrospectively assessed haemorrhoidectomies performed over a 4-decade period between 1935 and 1975 in an era when there were virtually no effective medical treatment options for Crohn’s disease and no specialized imaging. Corticosteroids were only introduced for ulcerative colitis in 1955 and generally thought to be of no value in the management of Crohn’s cases at that time with Azathioprine recommended by Bryan Brooke and Des Hoffmann in 1969 after they had tried it out on 6 patients, only 2 of whom had perianal fistulae [4]. Despite excellent outcomes after the excision-ligation operation for haemorrhoids in the 1930s being reported by Lockhart-Mummery [5], some publications that were contemporaneous with Ritchie showed even in non-Crohn’s patients an alarming list of complications after conventional haemorrhoidectomy [6].

However, these are data from a bygone era that today we cannot accept at face value as a major influence for our current surgical decision-making. Back then, this warning came almost as an edict from the world’s premier coloproctological facility. Today, we have manuals providing management guidelines, consensus criteria for the recording of inflammatory bowel disease (IBD) and perianal disease activity, routine imaging to define and track deep-seated and complex perirectal sepsis, a gamut of immunosuppressive treatments, and multiple centres with extensive coloproctological experience, specifically in the management of IBD and its complications.

My point here is that it is hard to know precisely what the quality/assurance of a 1930s–1970s excision-ligation haemorrhoidectomy actually looked like. We can also add on the fact that for this patient cohort, there was minimal or no anatomical knowledge of the perianal patho-anatomy and no medical treatment available for the cases presented. Once something is embedded into the literature and purveyed by the highest authority, it can be difficult to extricate. As coloproctologists, we each approach our complicated cases with an open mind, balancing knowledge of the evidence base with our sober judgment and individual experiences. However, this fairness of approach behoves us to recognize that the literature is produced at moments in history, and if it is to be incorporated into practice, it should be scrutinized with a modern eye.
